# Socioeconomic and race/ethnic disparities in observed park quality

**DOI:** 10.1186/s12889-016-3055-4

**Published:** 2016-05-12

**Authors:** Jessa K. Engelberg, Terry L. Conway, Carrie Geremia, Kelli L. Cain, Brian E. Saelens, Karen Glanz, Lawrence D. Frank, James F. Sallis

**Affiliations:** Family Medicine and Public Health, University of California, 3900 5th Ave Suite 310, San Diego, CA 92103 USA; Department of Pediatrics, Seattle Children’s Research Institute, University of Washington, 2001 Eighth Avenue, Suite 400, Seattle, WA 98121 USA; Perelman School of Medicine and School of Nursing, University of Pennsylvania, 801 Blockley Hall, 423 Guardian Drive, Philadelphia, PA 19104 USA; School of Community and Regional Planning, University of British Columbia, #433-6333 Memorial Road, Vancouver, BC V6T 1Z2 Canada

**Keywords:** Park quality, Disparities, Inequalities, Park facilities, Physical activity

## Abstract

**Background:**

Though park presence and access disparities are well studied for their associations with physical activity (PA), disparities in the availability and quality of amenities and facilities within parks have been infrequently examined.

**Methods:**

Five hundred forty-three parks from 472 block groups in the Seattle, WA and Baltimore, MD regions were audited using the Environmental Assessment of Public Recreation Spaces (EAPRS) to assess presence and quality (e.g., condition, cleanliness) of amenities (e.g., restrooms, seating) and facilities (e.g., fields, courts). General linear model regressions investigated Census 2000-derived neighborhood race/ethnicity and income main effect and interactive relationships with 7 park quality summary scores: 1) trails, 2) open space, 3) sports facilities, 4) PA facilities count, 5) PA facilities quality, 6) aesthetics, and 7) overall amenities, controlling for park size. The regions were analyzed separately due to differing race/ethnicity distributions.

**Results:**

In the Seattle region, neighborhood income was significantly negatively associated with sports quality score (*p* < .043), PA facilities total count (*p* < .015) and the overall amenities quality score (*p* < .004) (unexpected direction). In the Baltimore region, neighborhood race/ethnicity (percent White/non-Hispanic) was significantly positively related to the open spaces quality score (*p* < .011) (expected direction). A significant income-by-race/ethnicity interaction was found for PA facilities quality (*p* = .014), with high-percent minority neighborhoods having higher quality parks in high- vs. low-income neighborhoods, yet was opposite in mostly White/non-Hispanic neighborhoods. The other income-by-race/ethnicity interaction was for overall amenities quality score (*p* = .043), where scores in high-percent minority neighborhoods were best in high- vs. low-income neighborhoods. There was little difference in scores within mostly White or mixed neighborhoods by income.

**Conclusions:**

Patterns of association of neighborhood race/ethnicity and income with park qualities differed between regions. In the Seattle region, “equitable differences” were found, where lower income neighborhoods had better park quality on average. In the Baltimore region, park quality was more consistently negatively associated with income and race/ethnic diversity, and complex interactions of race/ethnicity by income were detected. These findings emphasize the need to explore other factors that may explain variations in park quality, like local policy, citizen involvement in park decision-making, park funding and allocation, sources of funding and park priorities.

## Background

Parks are a common location for leisure time physical activity [3, 8, 17, 21]. Studies have identified two categories of environmental correlates of physical activity within parks: 1) park proximity, i.e., the presence of a park nearby, and 2) the quality of parks, defined by the presence and quality of facilities and amenities [12, 20, 32, 35].

Given that parks are promising resources for physical activity, it is important to understand inequities in park access and quality that could contribute to disparities in leisure time physical activity [7] and health [27]. Park proximity has been explored as a protective factor for chronic disease. A study in Kansas City, MO found participants without a park nearby (i.e., within half a mile) were more than twice as likely to have 2 or more chronic health conditions, than those with a nearby park (Besenyi et al. [4]). The literature on potential disparities in park proximity has been largely cross-sectional and produced mixed results, with three prominent patterns of findings. The first pattern is disparities in the expected direction, where low-income and/or high-percent minority neighborhoods had fewer nearby parks [11, 19, 28]. The second pattern is opposite than the expected direction. Example findings include low-income and high-percent minority neighborhoods with greater access to parks than their counterparts [5, 39], mixed-race neighborhoods with the highest number of parks, regardless of income [1, 23], and low-income areas (often located in older parts of cities) with more parks than high- or medium-income areas, with no difference by race/ethnicity [38]. The third pattern includes no significant difference in park access in neighborhoods varying by income and/or racial/ethnic composition [29, 36].

Park proximity, or access, is only one component of parks that is relevant to physical activity. A study in Australia concluded the presence of public open spaces was not linked to cardiometabolic health, but rather the characteristics of the spaces were related to the health outcome [30], which strengthens the rationale for the present study. Therefore, other aspects of parks, including quantity and quality of amenities and facilities within parks, require examination. The limited research on objective measures of park quality show mixed directions of the relation between park quality and income and/or race/ethnicity. Suminski et al. [35] examined urban neighborhood parks and found parks in neighborhoods with a high percentage of racial/ethnic minorities had lower quality features and amenities than those in primarily white neighborhoods (controlling for income). Crawford et al. [10] found that high socioeconomic status (SES) neighborhoods had more amenities and positive aesthetics (e.g., trees, ponds, lighting) than low SES neighborhoods. A different study found that low-income areas had lower quality parks and that medium-income areas had more positive aesthetic features than both high- and low-income areas [38]. Kamel et al. [23] studied parks in a US-Mexico border region and identified disparities in park amenities and quality. They found no difference in park amenities by income tertiles, yet significantly more park quality concerns in the high foreign-born tertiles (i.e., high minority).

There are several gaps in park quality disparities research that the present study aimed to address. First, it is difficult to disentangle the effects of income and race/ethnicity because they are highly related. The present study examined race/ethnicity and income separately, as well as their interaction. Second, the lack of a common measure of park quality makes different studies difficult to compare. Though there is no consensus on measures of park quality, the present study used a systematically developed and evaluated direct observation instrument (EAPRS) that is often-used [9, 22, 31, 33]. Third, though most studies collected numerous variables related to park quality, some studies only used composite park quality scores in analyses [15, 38], while others analyzed the quality of specific facilities or amenities [35]. The present study included both composite and specific quality measures to provide a more comprehensive and granular assessment. Understanding disparities of specific features may provide information on which to base interventions to eliminate the disparities. The purpose of the present study was to examine the relationship of various aspects of park quality with the race/ethnicity and income of neighborhoods in which these parks are located.

## Methods

### Background

Present analyses used environmental park data from the Teen Environment and Neighborhood (TEAN) study, which was designed to improve understanding of the multi-level correlates of physical activity among adolescents, emphasizing environmental correlates, based on ecological models [34]. The Institutional Review Boards of San Diego State University and Seattle Children’s Hospitals approved the TEAN study.

### Study design & neighborhood selection

TEAN is a cross-sectional observational study conducted in the metropolitan regions of Seattle, WA and Baltimore, MD between 2008 and 2010. The study was designed to maximize variability of neighborhoods and participants based on their “walkability” characteristics and census-based neighborhood income. Census block groups in the study regions were identified and categorized into four neighborhood types (termed quadrants) formed by combinations of low versus high levels of built environment factors related to walkability and low- versus high-income, an indicator of SES.

A geographic information system (GIS) was used to generate the built environment variables, specifically ESRI ArcGIS v.9.2 and v.10 software. A 4-factor walkability index was used, based on GIS data, with walkability defined by higher net residential density, more mixed land use, more street connectivity (intersection density), and higher retail floor area ratio (an indicator of pedestrian-oriented design) [14]. High vs. low walkability was determined by a median split in each region [6]. The 928 TEAN participants were recruited from those block groups, and parks in these regions were identified.

### Park enumeration and selection

The research team first created a comprehensive list of parks in each study area. Parks were identified through various forms of digital and print lists, including local jurisdiction-supplied park lists, GIS shapefiles with park boundaries, websites, and commercial maps and listings. After the list was compiled, duplicates were removed based on name and addresses for a total of 1482 parks from the Seattle region and 1397 parks from the Baltimore region.

Up to three parks that intersected with or were within a 1 km buffer of the TEAN participant’s residence were audited. When more than three parks were present, the first was selected based on proximity to the residence and the other two were selected in order of size (i.e., the two biggest parks). They were then assigned to block groups based on the park address. The parks were audited with a systematic direct observation instrument (i.e., EAPRS; see below) by trained research staff for presence and quality of physical activity facilities and amenities. The study design and protocol for identifying parks did not include all parks in the areas or ensure a representative sample of parks from each region was studied. However, auditing all parks in the two regions was not feasible, and the present sampling approach ensured variability in neighborhood environments and demographics. Ultimately, 385 parks in the Seattle region and 335 in the Baltimore region were selected for audits. However, after raters went into the field, certain parks either did not exist (i.e., could not be found) or were not ratable (e.g., private, inaccessible). Therefore, audits were only conducted on 294 parks in the Seattle region and 256 parks in the Baltimore region, yet seven rated parks could not be matched with census data.

## Measures

### Census block group variables

Census block groups were selected instead of census tracts because they are the lowest level of census geography that has demographic data publicly available. The 2000 US Census data were used to provide information on the demographics of each block group identified as containing the address of a park enumerated for TEAN study participants. Because only the park address was used to assign the park to a block group, it is possible the park intersected with more than one block group and was missed. Census-derived variables considered in the present study were race/ethnicity (collapsed to reflect a mutually exclusive dichotomy of the proportion of the block group’s population, reporting only White/non-Hispanic race/ethnicity versus any other race/ethnicity), and median household income. Because demographics were assessed only for the block group that the park address fell within, it is possible that the parks intersected other block groups and the demographics of those block groups could have varied from the demographics of the block groups used.

### Environmental Assessment of Public Recreation Spaces tool (EAPRS)

EAPRS is a direct observation instrument to assess park environmental features and their quality, with documented inter-rater reliability [33]. Various versions have been used in other park quality studies [12, 22, 31], and some provided evidence of validity through association with physical activity [12, 22]. The EAPRS tool assessed whether a park had specific facilities and amenities present, as well as the quality of these facilities and amenities, most commonly their condition (e.g., intact, working as expected) and cleanliness (e.g., free of debris, graffiti). For example, if a basketball court was present with no nets and no painted lines, the court would be counted as a sports facility but would have a lower quality score.

### Terms

Park *features* were defined as either amenities or facilities. *Facilities* were considered places for physical activity, such as fields, courts, paths, places to swim, play structures, etc. *Amenities* were structural and not directly used for physical activity; for example, restrooms, picnic tables, water fountains, seating. *Aesthetics* refer to how the environment looked and included meadows, woods, landscaping, art, fountains, views, etc. *Quality* referred to the combination of the condition and cleanliness of a feature.

### Adaptations of EAPRS

Modules within EAPRS were used that were thought to be most relevant for adolescents (e.g., trails, paths, eating/drinking amenities, sports facilities, landscaping, general aesthetics, access-related features, athletic fields and other recreation centers). For example, the playground section was not used. (Version 7; available at: http://www.seattlechildrens.org/research/child-health-behavior-and-development/saelens-lab/measures-and-protocols). Parks were divided into 5 size categories: ≤1 acre, >1–5 acres, >5–10 acres, >10–50 acres and >50 acres.

### Procedures

EAPRS data were collected in 2009–2010 in Baltimore, MD region and in 2010 in Seattle/King County, WA region. Raters followed a detailed EAPRS protocol with directions, definitions, and photographs. All raters underwent extensive training from the same trainer, with certification, and ongoing evaluation of inter-observer agreement, feedback, and retraining as needed. Ratings took an average of 27 min in Seattle (range of 2–125 min) and 44 min in Baltimore (range of 2–180 min) to complete. Each rater completed up to 5 parks per four-hour day in the field, depending on travel distances. Inter-rater reliability ratings were completed for approximately 10 % of the surveys, across both sites.

### EAPRS Scoring

The original EAPRS total scoring was designed to account for both the presence/absence and quality of park features. The present study used a modified scoring procedure that included only facilities and amenities found to be correlated with observed physical activity in parks. The scoring was developed in a validation study of 40 San Diego parks using both EAPRS audits and direct observations of park users and physical activity (Geremia, Cain, Saelens, Conway, Gavand & Sallis: Developing short versions of a park quality audit. In preparation) (Table 1). These subscales and abbreviated EAPRS scores were used for the present analyses.

**Table 1:** Physical activity and non-physical activity scoring outcomes

Subscale	Total Points Possible	Description of items that are summed to create each subscale [points possible]
Trail Quality	4	Presence of paved [1], Quality of paved [1], Presence of unpaved [1], Quality of unpaved[1]
Open Space Quality	2	Presence of open space[1], Quality of open space[1]
Sports Facilities Quality	6	Presence of fields [1], Presence of courts [1], Presence of skate park [1], Quality of fields [1], Quality of courts [1], Quality of skate park [1]
PA Facilities Total Count	8	Sum of presence of: trail (paved and unpaved) [1], open space [1], sports facilities [3], pool [1], beach [1], and sidewalk [1]
PA Facilities Total Quality	16	Sum quality (plus presence) of trail (paved and unpaved) [2], open space [2], sports facilities [6], pool [2], beach [2], and sidewalk [2]
Aesthetics Total (Overall)	10	Quality (includes neighborhood condition) [1] Presence of: Meadows [1], woods [1], ponds [1], streams [1], fountains [1], views [1], historical markers [1], landscaping [1], art [1]
Amenities Total (Overall)	35	Neighborhood visibility [1] Presence of 17 items: Grills [1], picnic areas [1], restrooms [1], shelters [1], stages [1], parking lots [1], maps [1], seating [1], drinking fountains [1], vending [1], trash cans [1], entrances [1], bike racks [1], signs [1], event postings [1], telephones [1], wildlife areas [1] Quality of 17 items (if present): Grills [1], picnic areas [1], restrooms [1], shelters [1], stages [1], parking lots [1], maps [1], seating [1], drinking fountains [1], vending [1], trash cans [1], entrances [1], bike racks [1], signs [1], event postings [1], telephones [1], wildlife areas [1]

### Variables used

The present study analyzed a total of 543 parks from the Seattle and Baltimore regions, from a total of 472 block groups. The dependent variables from the EAPRS data were 7 park summary scores: 1) trail total quality score, 2) open space quality score, 3) sports facilities quality score, 4) physical activity facilities total count, 5) physical activity facilities total quality score, 6) aesthetics total score, and 7) amenities total score. Two independent variables were assigned to each park from census block group values: 1) percent White non-Hispanic and 2) median household income, from 2000 Census data.

### Analysis

Primary analyses using general linear model regressions were conducted, where each park quality measure was the dependent variable. Independent predictors tested were block groups’ proportion White/non-Hispanic and median household income (used as continuous variables in $10,000 increments for ease of interpretation) and their cross-product as the interaction term (*p* < .05 considered significant). Park size was included as a covariate due to the significant correlations between park size and most park quality variables in both regions, so as to not confound park size and park quality. Spearman correlations between census-level race/ethnicity and income, used because they are the most conservative, were 0.352 (*p* < 0.01) in Seattle and 0.275 (*p* < 0.01) in Baltimore regions.

Seattle, WA and Baltimore, MD regions are different in terms of race/ethnicity distribution. Seattle (*n* = 290) was over 70 % White non-Hispanic while Baltimore (*n* = 253) was only 53 % White non-Hispanic. Given the differences in race/ethnic distribution (Table 2), general linear model regressions were conducted separately for each region. These analyses were conducted in SPSS Version 22 using the General Linear Model (Univariate) procedure with census-based income and race/ethnicity entered as continuous variables, adjusting for park size as a continuous measure. B regression estimates, 95 % confidence intervals, and significance levels for income, race/ethnicity, and their interaction effects were tabled. To illustrate significant interactions, the mean predicted values from models with significant interactions were graphed for the independent variables recoded into tertiles to represent low (<34 % White/non-Hispanic), middle (≥34 to <73.7 %% White/non-Hispanic) and high (≥73.7 % White/non-Hispanic) race/ethnicity block groups and low (<$44,912), middle (≥$44,912 to < $66,453) and high (≥$66,453) income block groups. Interpretations were based on visual inspection of graphs.

**Table 2:** Means (SD) of predictors, outcomes and covariates related to park quality by site (*N* = 543)

	Seattle, WA region (*n* = 290 census block groups) Mean (SD)	Baltimore, MD region (*n* = 253 census block groups) Mean (SD)
Demographic Predictors		
Proportion White/non-Hispanic	0.71 (0.20)	0.53 (0.31)
Median Household Income	$58,157.30 ($23, 135.79)	$58,730.43 ($23,726.32)
Park quality scores		
Trail total quality score [0–4]	1.88 (0.65)	1.73 (0.68)
Open space quality score [0–2]	1.70 (0.21)	1.56 (0.21)
Sports Facilities quality score [0–6]	1.92 (0.73)	1.89 (0.66)
Physical Activity Facilities total count [0–8]	3.28 (1.53)	2.85 (1.33)
Physical activity Facilities total quality score [0–16]	5.07 (2.20)	3.98 (1.65)
Aesthetics total score [0–10]	2.13 (1.15)	1.53 (1.15)
Amenities total score [0–35]	9.71 (4.69)	7.94 (4.53)
Park covariate		
Size of park (acres) [1–14,000]	50.36 (284.22)	37.51 (6.61)

## Results

### Demographic predictors and park quality scores

Except for race/ethnicity, the sample of block groups included in the present study were similar across regions in terms of income and on most park quality outcomes (Table 2).

### Park Quality outcomes based on race/ethnicity and/or income

#### Seattle region

There were 3 main effects of neighborhood income on differences of park qualities. Median block group income was significantly negatively associated with sports quality score, physical activity facilities total count, and the presence and quality of amenities score, while controlling for park size. There were no significant race/ethnicity main effects or interactions (Table 3).

**Table 3:** Park Quality outcomes models assessing the main effects of Income and race/ethnicity and the interaction in the Seattle, WA region (*n* = 290)

*Variable*	*B*	*Confidence Interval*	*P-Value*
Outcome: Trail total quality score			
Median Income	0.002	(−0.045, 0.048)	.938
White non-Hispanic	0.238	(−0.276, 0.752)	.362
Income*Race interaction	--	--	--
Outcome: Open Space quality score			
Median Income	−0.010	(−0.025, 0.005)	.178
White non-Hispanic	0.029	(−0.130, 0.188)	.720
Income*Race interaction	--	--	--
Outcome: Sports Facilities quality score			
Median Income	−0.065	(−0.129, −0.002)	.043
White non-Hispanic	0.061	(−0.560, 0.682)	.847
Income*Race interaction	--	--	--
Outcome: PA Facilities total count			
Median Income	−0.017	(−0.192, −0.021)	.015
White non-Hispanic	−0.372	(−1.361, 0.616)	.459
Income*Race interaction	--	--	--
Outcome: PA Facilities total quality score			
Median Income	−0.100	(−0.228, 0.027)	.123
White non-Hispanic	−0.701	(−2.142, 0.740)	.339
Income*Race interaction	--	--	--
Outcome: Aesthetics total score			
Median Income	−0.040	(−0.106, 0.026)	.234
White non-Hispanic	0.463	(−0.298, 1.223)	.232
Income*Race interaction	--	--	--
Outcome: Amenities total score			
Median Income	−0.385	(−0.648, −0.121)	.004
White non-Hispanic	−0.239	(−3.277, 2.799)	.877
Income*Race interaction	--	--	--

Park size was a covariate in all models

Income is presented in units of $10,000

#### Baltimore region

**Fig. 1:**
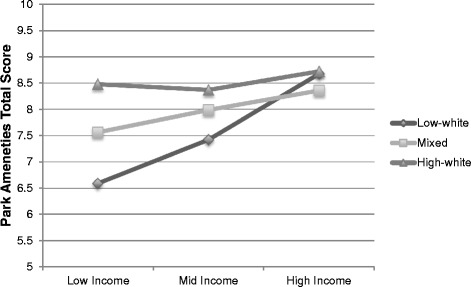
Amenities total score interaction by income and race/ethnicity (Baltimore). Tertile values were created at the following cut-points: low (<34 % White/non-Hispanic), middle (≥34 to <73.7 %% White/non-Hispanic) and high (≥73.7 % White/non-Hispanic) race/ethnicity block groups and low (<$44,912), middle (≥$44,912 to < $66,453) and high (≥$66,453) income block groups

Only 1 significant main effect was found: census block groups with higher-percent White/non-Hispanic populations had higher open spaces quality scores. There were 2 significant interactions of race/ethnicity and income with the physical activity facilities total quality score and amenities total score (Table 4). Within low-percent White/non-Hispanic block groups, the presence and quality of amenities appeared to be lower in low-income versus high-income block groups (Fig. 1), but this income gradation was not apparent in the high-percent White non-Hispanic or more evenly mixed race/ethnicity block groups.

**Table 4:** Park Quality outcomes models assessing the main effects of Income and race/ethnicity and the interaction in Baltimore, MD (*n* = 253)

*Variable*	*B*	*Confidence Interval*	*P-Value*
Outcome: Trail total quality score			
Median Income	0.035	(−0.020, 0.090)	.210
White non-Hispanic	0.215	(−0.293, 0.722)	.403
Income*Race interaction	--	--	--
Outcome: Open Space quality score			
Median Income	0.004	(−0.012, 0.020)	.639
White non-Hispanic	0.144	(0.034, 0.253)	.011
Income*Race interaction	--	--	--
Outcome: Sports Facilities quality score			
Median Income	−0.025	(−0.074, 0.025)	.326
White non-Hispanic	−0.054	(−0.397, 0.289)	.757
Income*Race interaction	--	--	--
Outcome: PA Facilities total count			
Median Income	−0.073	(−0.147, 0.001)	.053
White non-Hispanic	0.513	(−0.046, 1.072)	.072
Income*Race interaction	--	--	--
Outcome: PA Quality Score			
Median Income	--	--	--
White non-Hispanic	--	--	--
Income*Race interaction	−0.414	(−0.743, −0.084)	.014
Outcome: Aesthetics total score			
Median Income	0.003	(−0.060, 0.066)	.923
White non-Hispanic	0.255	(−0.225, 0.735)	.296
Income*Race interaction	--	--	--
Outcome: Amenities total score			
Median Income	--	--	--
White non-Hispanic	--	--	--
Income*Race interaction	−0.848	(−1.668, −0.028)	.043

Park size was a covariate in all models

Income is presented in units of $10,000

**Fig. 2:**
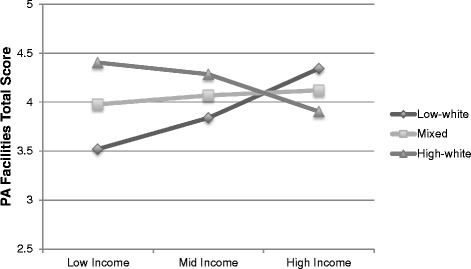
Physical Activity Facilities total quality score interaction by income and race/ethnicity (Baltimore). Tertile values were created at the following cut-points: low (<34 % White/non-Hispanic), middle (≥34 to <73.7 %% White/non-Hispanic) and high (≥73.7 % White/non-Hispanic) race/ethnicity block groups and low (<$44,912), middle (≥$44,912 to < $66,453) and high (≥$66,453) income block groups

The physical activity facilities total quality scores similarly appeared lower in low-income than high-income block groups within low-percent White/non-Hispanic block groups. The opposite trend was seen in the high-percent White/non-Hispanic block groups, where there were higher quality parks in low-income and lower quality parks in high-income areas. The quality of physical activity facilities in the mixed race/ethnicity block groups did not appear to differ by income (Fig. 2). For both interactions, the most notable variation in park quality scores occurred in the lowest-income block groups, with the lowest quality parks in high-percent minority block groups and the highest quality parks in high-percent White non-Hispanic block groups.

## Discussion

There were significant park disparities by income or race/ethnicity in each region. In final models of the Seattle region data, significantly higher quality parks were found in low-income block groups, with no significant differences by race/ethnicity. When poorer quality park scores were found in higher-income or higher-percent White non-Hispanic neighborhoods, they were considered evidence of what can be considered “equitable differences.” The term was adopted because providing better quality park features and amenities in areas usually considered disadvantaged could be a strategy to reduce health disparities. This unexpected pattern of “equitable differences,” based on the qualities evaluated by the EAPRS tool, suggests targeted investments in the Seattle region may have realized benefits for the most disadvantaged. Though there are inadequate data on park funding, King County (i.e., the Seattle region) has a countywide initiative to improve the equity across neighborhoods, including parks and open spaces, which went into effect in 2008 [26]. The present park data were collected in 2010, and the "equitable differences" found may be partially explained by the targeted initiative. A recent equity report that explored the existing characteristics related to the ordinance found “equitable differences” in that in 2000, 82 % of low-income and high-percent minority neighborhoods had park access compared to 65 % of high-income and high-percent White neighborhoods with park access. However, by 2010 these differences in park accessibility in the Seattle region had swung, where accessibility stayed the same for the low-income high-percent minority areas but grew to 95 % in the high-percent White high-income neighborhoods [2]. The findings from the present study may suggest that newer parks in high-income and high-percent White neighborhoods were not rich in the facilities and amenities measured here (e.g., more open green space and fewer other features), or that the targeted initiative was improving existing parks in low-income areas.

Though the Seattle region findings were in an unexpected direction, these findings are consistent with a subset of the park literature that also found “equitable differences” where low-income census tracts had more parks, with no differences in park availability/access by race/ethnicity [38]. Another potential explanation is that the wealthier, often suburban, areas are designed to be more isolated, and they may have private parks accessible to only those within a subdivision [5], which are not assessed in most studies. The Seattle findings indicate it is possible to avoid or correct for disparities in park quality, suggesting an important new line of inquiry to identify and further evaluate such mechanisms.

In contrast to the Seattle region, all significant findings (main effects and interactions), except one, from the Baltimore region were in the expected direction, with disparities showing lower park quality in lower-income and/or higher-percent minority areas. The main effect finding that areas with high-percent minority populations had lower open space park quality scores was consistent with some of the park quality literature. Suminski et al. [35] found that high-percent minority neighborhoods had lower-quality parks after controlling for income. A study of park funding in California assessed how funds from federal, state, special district, municipal, and nonprofit sources were allocated for parks and recreation facilities within Los Angeles County. They found funding from all sources favored middle-income communities over lower- and higher-income communities [18].

The current study was unique in that it also assessed interactions between race/ethnicity and income, and two significant interactions were found. The interactions in the Baltimore region for both the presence and quality of amenities outcome and the physical activity facilities quality outcome showed that the greatest disparities existed among high-percent minority neighborhoods, where high-income was associated with better quality parks compared to low-income.

However, the differences in park quality by income in high-percent White non-Hispanic neighborhoods were inconsistent across interactions. The interaction for physical activity facilities quality score showed “equitable differences,” where parks in high-percent White non-Hispanic neighborhoods appeared better in low-income versus high-income neighborhoods. This is similar to a finding from Wen et al. [39], who also found “equitable differences” in terms of park access. Jones et al. [19] also found a pattern across some regions in the US, including the Baltimore region, that low-income neighborhoods had greater access to parks but less access to other recreational facilities. Yet there were no interactions among the middle-income neighborhood parks by race/ethnicity. The lack of variance in park quality scores by race/ethnicity among middle-income neighborhood is consistent with the findings from Joassart-Marcelli [18] related to park funding in Los Angeles County, in that middle-income cities were favored, though it is unclear how generalizable the funding pattern is across regions.

It is important to consider the history of each region’s parks, specifically differences in the ages of the parks and their development. In *An Environmental Justice Inquiry in Baltimore, Maryland*, Boone et al. [5] explained the history of Baltimore’s parks from the mid-1800s, which included a focus on creating equal access to recreational space starting in the early 1900s. However, given the racial injustices at the time, this ultimately resulted in providing parks in predominantly White neighborhoods. After the post-WWII White flight movement, an extensive network of parks in Baltimore still existed, though the racial/ethnic make-up of the neighborhoods changed. Boone et al. [5] states, “In essence, the high access ratio for blacks is a hand-me-down from former white neighborhoods, a historical legacy of white privilege” (p. 783). Boone et al. [5] further argues that though blacks currently have greater access to parks within walking distance (≤400 m) than Whites, they have less park acreage available than Whites. Areas that were over 75 % White had about 53 acres per thousand, while areas over 75 % Black had <13 acres per thousand [5]. Beyond concern related to disparities in park size, it is likely that the quality of the parks’ features and amenities could be lower, particularly if urban neighborhoods and parks are older and not renovated. However, Boone et al. [5] did not have park quality data, which the present study added. The present study area included the City of Baltimore plus four counties, which could partially explain some of the findings due to the White flight and suburban sprawl Boone discusses.

Though parks existed much earlier in the Seattle region, it was not until the 1968 Forward Thrust era that the local government started to focus on acquiring park sites and constructing facilities, according to the King County (i.e., Seattle region) archives on the history of their parks (King County Archives Exhibits [24]). As of 2006 the Seattle region had almost 15 acres per thousand population [25, 35]. Data on the distribution and equity of park and open space across the Seattle region were unavailable; however, as mentioned above, an Equity and Social Justice Initiative started in 2008. This initiative encompasses Ordinance 16948, which includes a section devoted to parks and natural resources, stating that they must “…provide access for all people to safe, clean and quality outdoor spaces, facilities and activities that appeal to the interest of all communities” ([2], p. 84).

Given the relative age differences of the regions, as well as the differences in race/ethnicity composition and influence of segregation, particularly in the Baltimore region, it is likely that the community dynamics are different, which would affect funding and prioritizing of parks in community agendas. However, it would be useful to conduct more extensive historical research to shed light on the origins of regional patterns related to park access and quality.

### Limitations

The present study was limited because the data were cross-sectional, the parks and census block groups were not randomly selected, only the park address was used to match it with a block group, no information on park funding allocation was collected and there were no measures of park use or physical activity in parks. Using the crude racial/ethnic categories of percent White non-Hispanics vs. percent minority prevented exploration of disparities among areas dominated by specific racial/ethnic subgroups that may have different patterns. It would have required a much larger study to assess specific race/ethnic group differences at the block group level. Another limitation was that though multiple comparisons were made during the analyses, there was no adjustment for Type 1 error. The rationale was that exploring interactions was a primary aim, and there is reduced power for detecting interactions. Additionally, whereas EAPRS assesses many objective aspects of park environments, the perceived safety of park users or potential park users may be an important unstudied factor that differs between parks in contrasting regions.

The TEAN study design that stratified on walkability and income is an appropriate design for the present study because it ensured variability in environments and demographics, yet it did not ensure a representative sample of parks. Because only the park address was used to assign the park to a block group, it is possible the park fell into more than one block group, or that the demographics of those other block groups could have varied from the demographics of the block group used in the current study. Given the wide geographic scope of the study, it would not have been feasible to conduct the time-consuming direct observations of all parks in the regions. As such, the current study is still a valuable contribution to the objectively measured park quality literature by highlighting regional differences in patterns of disparities.

## Conclusion

The inconsistent findings between the two regions echo the mixed findings in the existing park quality literature [10, 23, 32, 35, 38]. Despite using more refined methods, including objective measures of park quality validated by correlations with physical activity and assessing for interactions between race/ethnicity and income, there were no generalizable patterns of disparities in park quality across the two regions.

One potential explanation for the inconsistencies between findings in both the existing park quality literature and the current study is that there are real differences in park quality patterns across cities due to local community dynamics including policies, funding, and citizen involvement related to park planning and governance. A recent study by Jones et al. [19] also found that patterns by race/ethnicity and income related to park and recreation center access across 6 US cities varied by region. Their conclusion that these differences may be explained by regional differences in policies and variation in resource availability is consistent with the implications of the present study. Though limited, the data on the allocation of park funding suggests that differential funding heightens disparities, at least in one region of the US [18, 37]. For example, a policy brief that summarized relevant findings from the cities in Los Angeles County concluded parks and recreation facilities are primarily funded using localized sources, in that almost 75 % of the funding came from municipal governments [37]. The differences between local cities’ funding towards parks was highly discrepant, where per capita total expenditures ranged from $0.55 to $593. In the lowest-income cities, capital spending was 12.6 % of parks and recreation expenditures compared to 26.1 % in the highest-income cities [18]. When larger cities of the US were compared, Seattle spent the most per capita on parks, which was four times more than what Baltimore spent [16]. However, these numbers are dated as they used 1990 data and are representative only of the cities assessed but not the overall regions or counties. No data could be located specific to park funding of the overall Baltimore or Seattle regions or counties, but given the findings from the current study and the Seattle ordinance discussed earlier, it is possible that the Seattle region is providing more park funding for low-income areas.

Future research should replicate the use of validated direct observation measures of park quality in many more locations, particularly given the scoring of EAPRS that is based on areas in parks where physical activity occurs. There are currently limited data on high-risk populations’ observed park-based physical activity and the impact of facilities and amenities. A study in Australia assessed the influence of observed park quality and park size on use of parks, and found that larger, more attractive (i.e., high quality) parks were associated with significantly higher levels of walking [15]. Floyd et al. [13] found facilities and amenities appear to be related to park use for all race/ethnic groups. However, Hispanic and Black park users were more frequently observed in vigorous physical activity, so these groups may have different preferences for facilities than other race/ethnic groups. Another study also suggested different patterns of use and facility preference across race/ethnic group. Park-based physical activity among White non-Hispanic participants was positively associated with presence of 4 specific facilities (playgrounds, splash pad, fitness station, and skate park), but among Black participants park-based physical activity was positively associated with only 1 specific facility (basketball court) [20]. There is a distinct gap in the knowledge base regarding how various population subgroups use parks and what kinds of facilities and indicators of quality are most important to promote park-based physical activity.

Findings from the present study highlighted disparities by race/income in the Baltimore region and “equitable differences” in park quality by income in the Seattle region. Given these findings, future research should assess differences in park quality in enough regions and jurisdictions to allow exploration of the role of other factors, such as local policies, citizen involvement in park decision making, park funding and allocation, sources of funding, and park priorities.

From a policy and decision making context, it would be most helpful to know the relative cost and benefits of investments in different types of features in parks within different demographic contexts. Research is needed that extends the physical activity benefits from investments in parks to reduced chronic disease incidence and health care cost savings. It is plausible that investments in recreational infrastructure in parks and improved green infrastructure may have mortality benefits and increase productive life years. Comparing amount spent on parks with amount saved through morbidity and mortality within different sociodemographic contexts may help to justify, prioritize, and target future expenditures on parks. Because findings from studies of park disparities have been inconsistent, the research priority should be to better understand the local dynamics that might account for local differences in park quality disparities.

### Consent

IRB approval was received from San Diego State University, which included consent and assent from all participants. However, the current study did not use any data from participants and instead used block group Census-derived data to obtain demographic information (e.g., race/ethnicity and income). Park quality data was collected via direct observation and did not involve participants.

### Availability of data and materials

At this time, the authors are unable to share the data on the grounds of confidentiality and risk of losing anonymity. However, it is possible that some of the data can be made available by request.
